# Microbial Biosurfactant: A New Frontier for Sustainable Agriculture and Pharmaceutical Industries

**DOI:** 10.3390/antiox10091472

**Published:** 2021-09-15

**Authors:** Ajay Kumar, Sandeep Kumar Singh, Chandra Kant, Hariom Verma, Dharmendra Kumar, Prem Pratap Singh, Arpan Modi, Samir Droby, Mahipal Singh Kesawat, Hemasundar Alavilli, Shashi Kant Bhatia, Ganesh Dattatraya Saratale, Rijuta Ganesh Saratale, Sang-Min Chung, Manu Kumar

**Affiliations:** 1 Agriculture Research Organization, Volcani Center, Department of Postharvest Science, Rishon Lezzion 50250, Israel; ajaykumar_bhu@yahoo.com (A.K.); arpanbiotek@gmail.com (A.M.); samird@volcani.agri.gov.il (S.D.); 2 Centre of Advance Study in Botany, Banaras Hindu University, Varanasi 221005, India; sandeepksingh015@gmail.com (S.K.S.); dharambhu@gmail.com (D.K.); prempratapsingh31@gmail.com (P.P.S.); 3 Department of Botany, Dharma Samaj College, Aligarh 202001, India; ckantop@gmail.com; 4 Department of Botany, B.R.D. Government Degree College, Sonbhadra, Duddhi 231218, India; vermahariom87bhu@gmail.com; 5 Department of Genetics and Plant Breeding, Faculty of Agriculture, Sri Sri University, Cuttack 754006, India; mahipal.s@srisriuniversity.edu.in; 6 Department of Bioresources Engineering, Sejong University, Seoul 05006, Korea; alavilli.sundar@gmail.com; 7 Department of Biological Engineering, College of Engineering, Konkuk University, Seoul 05029, Korea; shashibiotechhpu@gmail.com; 8 Department of Food Science and Biotechnology, Dongguk University, Seoul 10326, Korea; gdsaratale@gmail.com; 9 Research Institute of Biotechnology and Medical Converged Science, Dongguk University, Seoul 10326, Korea; rijutaganesh@gmail.com; 10 Department of Life Science, College of Life Science and Biotechnology, Dongguk University, Seoul 10326, Korea; smchung@dongguk.edu

**Keywords:** biosurfactants, critical micelle concentration (C.M.C.), antioxidant, microorganism, soil quality, plant disease management

## Abstract

In the current scenario of changing climatic conditions and the rising global population, there is an urgent need to explore novel, efficient, and economical natural products for the benefit of humankind. Biosurfactants are one of the latest explored microbial synthesized biomolecules that have been used in numerous fields, including agriculture, pharmaceuticals, cosmetics, food processing, and environment-cleaning industries, as a source of raw materials, for the lubrication, wetting, foaming, emulsions formulations, and as stabilizing dispersions. The amphiphilic nature of biosurfactants have shown to be a great advantage, distributing themselves into two immiscible surfaces by reducing the interfacial surface tension and increasing the solubility of hydrophobic compounds. Furthermore, their eco-friendly nature, low or even no toxic nature, durability at higher temperatures, and ability to withstand a wide range of pH fluctuations make microbial surfactants preferable compared to their chemical counterparts. Additionally, biosurfactants can obviate the oxidation flow by eliciting antioxidant properties, antimicrobial and anticancer activities, and drug delivery systems, further broadening their applicability in the food and pharmaceutical industries. Nowadays, biosurfactants have been broadly utilized to improve the soil quality by improving the concentration of trace elements and have either been mixed with pesticides or applied singly on the plant surfaces for plant disease management. In the present review, we summarize the latest research on microbial synthesized biosurfactant compounds, the limiting factors of biosurfactant production, their application in improving soil quality and plant disease management, and their use as antioxidant or antimicrobial compounds in the pharmaceutical industries.

## 1. Introduction

The rapid industrialization and rising global population excavate the challenges of food security and environmental management. Moreover, the changing climatic conditions, such as rising temperature, irregular rainfall, and biotic and abiotic stress factors adversely affect agricultural productivity. In addition, an eruption of new pests, pathogens, or plant diseases are some primary concerns for the agronomist, researchers, and scientific community. Indeed, a larger population of developed and developing countries rely on chemical pesticides or agrochemicals for pathogen control or plant disease management. Nevertheless, the undistributed and continuous use of agrochemicals results in the deposition of toxic chemical residue in the food, low nutrient quality, and the emergence of pesticide-resistant pathogens.

Additionally, the deposition of agrochemicals adversely affects the texture, nutrient quality, or the native microflora of the soil and also leads to environmental challenges via polluting soil and water ecosystems [1]. However, to mitigate these challenges, in the last two decades, microbes and their products have been frequently utilized to enhance agricultural productivity and crop yield or mitigate toxic and hazardous environmental contaminants. Moreover, the ubiquitous nature of microbes, easy cultivation methods, cost-effectiveness, and low or even no toxic effect on the surrounding environment makes them most preferable in the various fields for sustainable growth and production.

**Figure 1:**
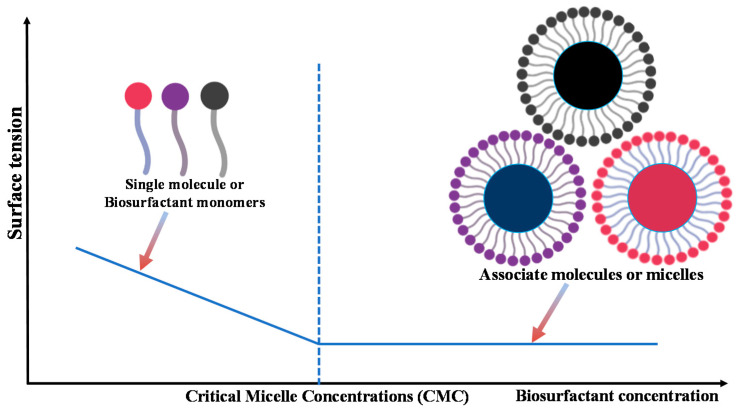
Critical micelle concentration (CMC) and micelle formation of biosurfactant monomers.

Biosurfactants are one of the latest explored microbial produced/synthesized biomolecules, and are frequently utilized in various agricultural, waste management, or pharmaceutical industries as raw materials, for the lubrication, wetting, foaming, emulsions formulations, or stabilizing dispersions [2,3]. The term biosurfactant has been referred to as the surface-acting agents that can improve surface–surface interactions through forming micelles produced by the natural source of origin, such as plants, microbes, and animals [4,5]. In addition, biosurfactants have been used during the applications to reduce the interfacial surface tension between solution and the surface, or air/water or oil/water interfaces [6]. In other aspects, the addition of surfactants into an oil/water or water/air system causes a reduction in the surface tension up to a point at which surfactants form structures, such as micelles, vesicles, and bilayers; usually, this critical point is known as critical micelle concentration (C.M.C.) (Figure 1).

However, in a combined mixture or after the addition of surfactant in the water and oil mixture, the surfactant resides at the oil/water interface and forms emulsions, which confer excellent emulsifying, foaming, and dispersing capacities. This makes surfactants one of the most versatile chemicals for industrial processes [7]. The most commonly used surfactants are of chemical origin, but their toxic nature, low degradation rate, and high persistence power limit their frequent use in the food, cosmetics, and pharmaceutical industries [8]. Surfactants of microbial origins have several advantages over synthetic or chemical surfactants: higher temperature tolerance, stability in pH variation, high salinity tolerance, higher degradation rate, less toxicity, and better selectivity [9,10].

**Figure 2:**
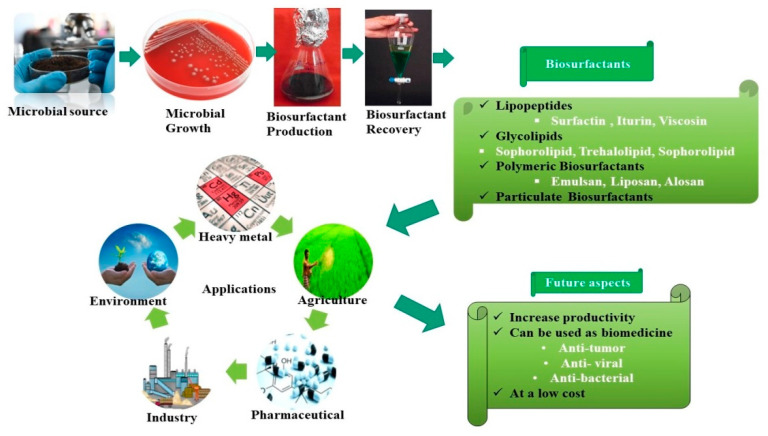
Schematic representation of biosurfactant production utilizing microbial resources and their potential applications.

Biosurfactants are usually composed of amphipathic molecules that have both hydrophilic and hydrophobic constituents. The hydrophilic compounds generally consist of positive, negative, or amphoteric charged ions, whereas the hydrophobic compounds are made up of a long chain of fatty acids [11]. Irrespective of their chemical counterparts, biosurfactants are generally classified based on molecular weight (low or high), critical micelle concentration (C.M.C.), microorganism produced, and their mode of action. Glycolipids, phospholipids, and lipopeptides are the most commonly reported low molecular weight; however, high molecular weight biosurfactants are composed of polysaccharides, lipopolysaccharides, and a complex mixture of biopolymers. A detailed classification and some common examples are illustrated in Table 1 and Figure 2.

The low molecular weight of microbial and synthesized biosurfactants offer excellent capability for reducing surface tension; however, high molecular weight is associated with the ability to make a stable emulsion [7,12,13]. The eco-friendly and multifunctional attributes of biosurfactants are considered the surfactant of the next generation and are frequently utilized in various industries worldwide. According to a published report, the market size of biosurfactants is expected to increase by 0.8% of the compound annual growth rate (CAGR) in the forecast period of 2020 to 2025. It will be expected to reach about USD 1446.5 million by 2025 from the USD 1403.1 million reported in 2019 (Global Biosurfactant Market 2020 by Manufacturers, Regions, Type, and Application, Forecast to 2025).

For sustainable agricultural practices, biosurfactants have been used to improve soil quality by degrading toxic and hazardous contaminants or making trace elements available in the soil, and are frequently utilized as antagonistic molecules against pests/pathogens or plant diseases. Surfactants produced by microbial strain possess antimicrobial properties, which effectively inhibit pathogen growth. In several cases, it protects the plant from pathogen infection via stimulating the plant immune system [14]. Furthermore, utilization of biosurfactants showed additional benefit in the plant through enhancing growth promotion. Additionally, the native microflora of the soil or plant system uses these biosurfactant molecules as a source of energy for regulating plants’ physiological parameters and maintaining the plant system’s health and quality.

Moreover, nowadays, in pharmaceutical industries, biosurfactant molecules are broadly used as antioxidants, antimicrobial and anticancerous agents, raw materials, or emulsifying or dispersing agents. Thus, the application of biosurfactants in treating human ailments is cost-effective and safe from toxic side effects. In the present review, we summarize the latest aspect of biosurfactant synthesis from microbial sources, limiting factors of biosurfactant production. In addition, it also discussed the potential application of biosurfactants in sustainable agriculture, specifically their role in improving soil quality or plant pathogen management and pharmaceutical industries as an antioxidant or antimicrobial molecule.

## 2. Microorganisms and Biosurfactants

Currently, numerous microbial strains of bacteria, fungi, and yeasts have been reported for the efficient production of biosurfactants. However, the quality and quantity of biosurfactants depend on several factors, including the type of microorganism, media supplements, nature of the substrate, and different intrinsic and extrinsic factors at the time of microbial culture growth [15,16]. The selection of microbial strain is the primary step of biosurfactant production. However, biosurfactant synthesis in the microbial strain is carried out either intracellularly or extracellularly during the exponential or stationary phase of growth, when the nutrient conditions are limiting [17]. The nature of biosurfactants also depends on microorganisms’ source and isolation strategies; for instance, a strain isolated from a contaminated site is considered a suitable choice for the degradation of that particular contaminant. The probable reason for this concept is that the isolated microorganism can use that contaminant as a source of energy or substrate, where other microorganisms or non-surfactant-producing microorganisms cannot survive [18].

Furthermore, biosurfactants play a physiologic role in increasing the bioavailability of hydrophobic molecules involved in cellular signaling or differentiation processes and facilitate the consumption of carbon sources present in the soil [19]. Indeed, the physiological aspect of biosurfactant production in the contaminated site is not clearly understood but considered for enhancing the nutrient uptake from the hydrophobic substrate, biofilm formation, and cellular motility by reducing the surface tension at the phase boundary [7]. The development of rapid and reliable methods for the isolation and screening of microbial strains and further evaluation of role in emulsification, reducing interfacial or surface tension are critical factors during the exploration of biosurfactant molecules [20]. In early 1941, Bushnell and Hass [21] reported biosurfactants produced by the bacterial strain *Corynebacterium* simplex and *Pseudomonas* grown in the minimal media containing kerosene, mineral oil, or paraffin [22]. Then, numerous microbial strains, including bacteria, fungi, and yeast, have been reported for efficient biosurfactant production. Details of microbial strains and their synthesized biosurfactants are illustrated in Table 2A–C.

## 3. Factors Affecting Biosurfactant Production

Traditionally, most biosurfactant-producing bacterial strains have been isolated from petroleum/oil-contaminated soil or fermented food, but nowadays, microbial isolates are screened from various sources. The production of biosurfactants started with the growth, identification, and characterization of microbial strains. The growth conditions of the cultures should be maintained according to the sample sites. However, the methodology, substrate, and purification process should be cost-effective for biosurfactant production at a commercial or industrial scale. According to a published report, 10–30% of the total cost accounted for raw materials during biosurfactant production [64], while up to 60% of the total cost has been spent on the downstream or purification processes [64,65]. The media components of the microorganism play an essential role in biosurfactant production and significantly impact the production cost. Carbon (C) and nitrogen (N) sources in the media are an essential requirement for microbial growth [66]. The type, amount, and ratio of carbon and nitrogen in the media directly affect microbial growth and biosurfactant production in both laboratories and large-scale industrial fermenters [67]. In most studies, glucose, sucrose, and glycerol are used as carbon and yeast extract, while NaNO_3_, urea, and soya broth have been used as a nitrogen source in the media [68,69]. For instance, an abundance of carbon sources and limiting nitrogen conditions are preferred for optimum biosurfactant production. For example, the ratio of C: N ≈ 20 has been found most favorable for *Pseudomonas* sp. [70]. In a study, Onwosi and Odibo [71] evaluated the role of carbon and nitrogen source, on the rhamnolipid production by the strain *Pseudomonas nitroreductase* and recovered 5.28 and 4.38 gL^−1^ of rhamnolipid using glucose as a carbon and sodium nitrate as a nitrogen source respectively.

Furthermore, the highest yield of 5.46 gL^−1^ was observed when the ratio of C: N (glucose/sodium nitrate) was 22. Thus, the selection of media sources has a significant impact on biosurfactant production. A detailed survey on the utilization of carbon and nitrogen sources and their implications for biosurfactant recovery has been described in Table 3. 

The production cost of biosurfactants largely depends on the media source, primarily the carbon and nitrogen sources. Therefore, in the recent past, a range of new and novel resources, such as residual waste products of the food industry, e.g., frying oil, distillery, molasses, and vegetable- and plant-derived oil, has been trailed in the media as a carbon and nitrogen source as single or together with the stabilized resource. The utilization of these products can cut or reduce the cost of biosurfactant production [88,89,90]. The use of vegetable oil and hydrocarbon-based substrates appear as economical and profitable substrates for large-scale biosurfactant production, especially from *Pseudomonas*, *Bacillus,* and *Candida* sp. [91].

There are numerous reports available in biosurfactant production using different nutritional sources and limiting environmental factors. For example, Agarwal and Sharma [92] utilized different C sources, such as glycerol, molasses, rice water, cheese whey, potato peels, and glucose, to evaluate their impact on biosurfactant production. They observed similar biosurfactant activity, using molasses and glycerol sources, and biosurfactants were produced using a glucose source. In addition, the utilization of NH_4_Cl, NH_4_NO_3_, and NaNO_3_ as a nitrogen source yielded good results. Similarly, Al-Bahry et al. [93] recovered 2.29 ± 0.38 gL^−1^ of biosurfactant, using date molasses as a carbon source from the strain *Bacillus subtilis* B20, which had the capability to reduce surface tension and interfacial tension from 60 to 25 mN m^−1^ and 27 to 5.02 mN m^−1^, respectively. In addition, biosurfactants showed stability against a wide range of temperatures, pH variations, and salt concentrations. Hentati et al. [94] reported 50 mgL^−1^ of biosurfactant production by the strain *Bacillus stratosphericus* FLU5 using residual frying oil as a carbon source. At this concentration, the surface tension of the water was reduced from 72 to 28 mN m^−1^. Similarly, Souza et al. [95] reported biosurfactant production by the strain *Wickerhamomyces anomalus* CCMA. Under optimized culture conditions, various amounts of biosurfactant has been recovered from the yeast strain using different energy resources, such as yeast extract (4.64 gL^−1^), ammonium sulfate (4.22 gL^−1^), glucose (1.39 gL^−1^), and olive oil (10 gL^−1^). However, the highest yield of biosurfactant was recovered from the 24-hour-old culture. Additionally, the biosurfactant remained stable even at a higher temperature of 121 °C, NaCl concentrations of 300 gL^−1^, and pH ranges of 6–12. A brief survey of biosurfactant production using alternative carbon and nitrogen sources and their impact on yield oand properties has been described in Table 4.

Besides nutrient sources, the production of biosurfactants depends on several factors, such as incubation time, incubation temperature, pH of growth culture, and the speed of rotation rate of shaking incubator, which directly affect the microbial growth and biosurfactant production. In one study, Achim et al. [110] evaluated the biosurfactant production potential of *Azotobacter chrococcum* under controlled nutritional and environmental conditions. The highest 68% of surface tension and emulsification index (EC24) was observed at pH 7. Sunflower oil and heavy oil 150 had shown the best response among different carbon sources and accounted for 76.6% and 74.1% of E.C. 24, respectively. However, higher EC24 was recorded after supplementing yeast extract (83.3%) and (NH4)_2_SO_4_ (80%) among different nitrogen sources. The optimum recovery of biosurfactant was achieved from 4 days old culture incubated at 30 °C, in a shaking incubator at 150 rpm.

Similarly, Joaad and Hassan [111] evaluated the biosurfactant potential of yeast strain Candida guilliermondii using the VITTEK2 compact system under controlled environmental and nutritional conditions. The maximum EC24 was 70% observed at pH 4 and 75% at 30 °C. However, sesame oil and heavy oil 150 were shown to give the best response when used as a carbon, along with addition of NaNO3 as nitrogen source. Additionally, the shaking incubator at 150 pm resulted in higher emulsifier production on the 7th day of culture growth.

## 4. Biosurfactant Applications in Improving the Soil Quality

The growth and productivity of the crop ecosystem rely on the availability and presence of an optimum concentration of micro- or macronutrients in the soil. Trace elements present in the soil directly influence the physiological processes of the plant. Indeed, deficiency or excess availability of these elements led to various diseases and poor quality of plant growth. The ongoing changing climatic condition, rising global temperature, variation in soil pH, increase in salinity, or deposition of environmental contaminants adversely affect the efficacy of trace elements in the soil, resulting in poor availability to the plants, which ultimately results in lower crop production and poor food quality [112].

The addition of biosurfactants in the soil significantly enhances the availability of micronutrients in the mineral deficient soil through various processes. The addition of surfactant makes a complex with the metal ion, which, through biochemical processes such as oxidation reduction, adsorption, and deadsorption, increases their bioavailability or concentration in the soil [113]. In detail, an anionic charge of surfactant binds with the cationic charge of the metal and forms a complex; through this way, it acts as a sequestering agent and performs desorption of the soil [114]. However, in contaminated water, the flushing of water through soil can remove metal surfactant complex from the soil because of the strong electrostatic interaction between the opposite charge ion of the metal and surfactant, resulting in metal mobilization in the water [115,116]. The application of biosurfactants can also help mitigate the challenge of soil alkalinity, which is considered one of the paramount factors of micronutrient deficiency in the soil. The addition of biosurfactants makes the metal–biosurfactant complex available by removing or unbinding the metal from the soil complex [117]. During this interaction, the bond strength of the metal–biosurfactant interaction is much higher than the metal–soil interaction, which further desorbed the metal–biosurfactant complex from the soil matrix to the soil solution, due to a lowering of the interfacial tension, resulting in the availability of trace elements to the plant roots [115,118]. The addition of surfactant reduces the interfacial tension between the metal and soil, forms micelles, and transfers them to the root zone interface. 

**Figure 3:**
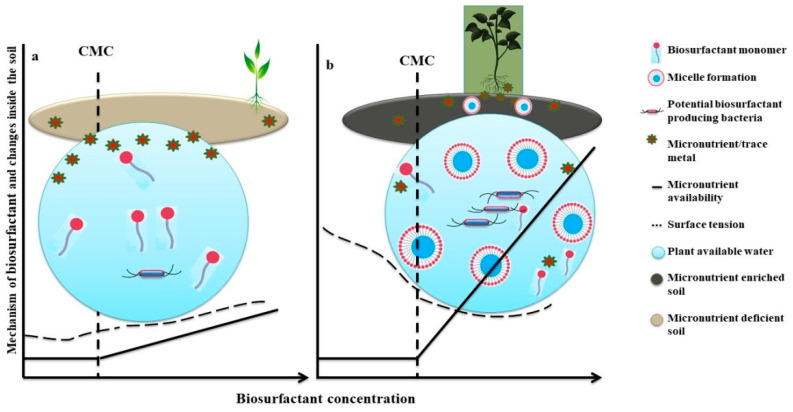
Impact of micronutrient deficiency on plant growth and soil quality on a micronutrient-deficient soil. (**a**) Mechanisms of biosurfactant application enhancing micronutrient availability in micronutrient-deficient soil to the plant, soil quality, and related water quality through increasing nutrient solubility at a fixed concentration of biosurfactant molecule. (**b**) C.M.C. at which there is a sudden increase in metal solubility in the system. Figures are adapted and modified from Mulligan [13] and Singh et al. [114].

The use of biosurfactants in the agricultural field to improve or enhance the availability of micronutrients to the soil is the new approach and is, nowadays, broadly practiced in different parts of the world [115]. The amphipathic nature of biosurfactants can reduce the interfacial tension between two immiscible liquids and enhance the solubility of organic and inorganic components [119,120]. In the agricultural process, different biosurfactants are reported to decrease the interfacial surface tension between the solid surfaces and the trace metal cations, resulting in increased solubility and mobility of trace elements [121] (Figure 3). 

For instance, Sheng et al. [122] reported that the strain *Bacillus* sp. J119 has biosurfactant capability, which significantly enhances the uptake of trace elements and promotes the growth potential of canola maize, sudangrass, and tomato. Furthermore, Stacey et al. [123] reported the formation and plant uptake of lipophilic metal-rhamnolipid complexes that facilitate the Cu, Mn, and Zn uptake and movement in *Brassica napus* and *Triticum durum* roots.

In addition, the application of biosurfactants, as they have a microbial origin, significantly modulates plant growth via synthesizing phytohormones and inducing resistance. Therefore, the efficiency and availability of micronutrients in the soil to the plants might be increased, either due to bioaugmentation of biosurfactant-producing bacteria [124]. The application of biosurfactants also influences the native microflora of the plants or soil, directly or indirectly responsible for growth promotion, mitigating biotic and abiotic stresses, and removing contaminants from the soil or plant roots. In one study, Liao et al. [125] used the pot experiment with maize to examine the effect of biosurfactant (rhamnolipid and lecithin) and chemical surfactant (Tween 80). After application in the crude oil-contaminated soil, it did not significantly affect the maize biomass, while rhamnolipid and lecithin application enhanced the microbial population, resulting in increased petroleum hydrocarbons from the contaminated soil. March-Mikołajczyk et al. [126] reported on the Glycolipid produced by endophytic bacterial strain *Bacillus pumilus* 2A, which after application significantly improves the growth of bean, radish, and beetroot. Chopra et al. [127] evaluated different rhizobacterial strains of tea, in which one of the strains, *Pseudomonas aeruginosa* RTE4, produced di-rhamnolipid biosurfactant and showed multiple growth-promoting traits as well as fungicidal activity. Similarly, Alsohim et al. [128] reported that the viscosin produced by *Pseudomonas fluorescens* S.B.W. 25 helps with spreading the motility, which facilitates the colonization efficacy of microbial strain and showed growth promotion potential.

## 5. Biosurfactant Application in Plant Disease Management

Plant disease causes a significant reduction in agricultural commodities during pre-or post-harvest conditions and is considered a severe threat to food security for the rising global population. It has been estimated that approximately 30% of the total agricultural production is destroyed due to various plant diseases and pathogen attacks, either during pre- or post-harvest storage conditions [129]. However, to manage phytopathogen and plant diseases, farmers most often rely on chemical pesticides. Nevertheless, the undistributed and continuous use of chemical pesticides during crop production led to various adverse consequences, such as poor food quality, soil and water pollution, pest resistance, effects on natural microbiota, and severe health issues to consumers. Moreover, various microbial biocontrol agents, including bacteria, fungi, and yeasts, have been frequently utilized to manage plant diseases. They showed an effective response against phytopathogen growth, fruit quality maintenance, or storage life enhancement.

Agrochemicals are preferred more frequently than other crop protection or plant disease management resources because of their easy availability and quick response. Nevertheless, traditional formulation and low dispersion capacity on the target site, either the plant surface or the pathogen, led to lower efficacy and environmental pollution. According to the report, it has been estimated that only about 0.1% of the total applied pesticides reach the target organisms, and the remaining bulk contaminates the surrounding [130,131].

**Figure 4:**
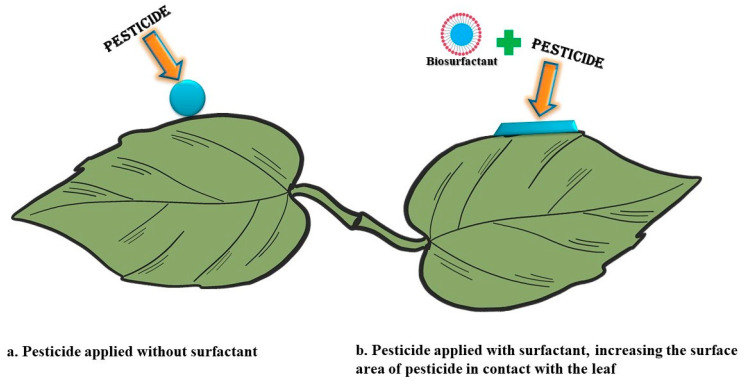
Depiction of the effect of surfactant on leaf surfaces. (**a**) Pesticide applied without surfactant; (**b**) pesticide applied with a surfactant, increasing the surface area of the pesticide in contact with the leaf. Figure adapted from Jibrin et al. [138].

In common practice, a pesticide is either sprayed directly on the plant and surfaces or the plant is sometimes dipped into the pesticide solutions. Still, drift does not reach the target site and shows poor efficacy against disease management [132]. However, nowadays, to improve the effectiveness of pesticides applications, the delivery mechanism has been upgraded via adding surfactants, nano-based formulations, and improved spraying technology [133,134]. In general, during pesticide application, surfactants have been used as an additive or adjuvants and mixed with pesticides that help in dispersion, emulsification, better spreading, or increasing the contact area with the plant surface, which enables the pesticides to better reach the target pests or target organisms [135] (Figure 4). 

However, after mixing and applying biosurfactants with pesticides, care should be taken, because surfactant application may harm the non-target phytobiome and plant physiological process [136]. Moreover, the enhanced permeability of pesticides may lead to increased residue levels in plant tissue and fruits [137]. Therefore, selection and the concentration of surfactants are prime factors that need to be considered for better disease management strategies.

Currently, a range of chemically synthesized surfactants, such as Triton X-100, Cohere, Agral 90, Silwet L-77, and Tween 20, are some of the most common synthetic surfactants used for plant disease management, and they have displayed improved insecticidal potential during in vitro and in vivo applications [138,139]. However, due to its chemical and toxic nature, direct application on the plant surface was avoided. Unlike synthetic surfactants, which are usually used as adjuvants, most biosurfactants have been directly applied on the plant surface for disease management [140,141], and nowadays, continuously new biosurfactant-producing microorganisms have been screened and explored for their optimum recovery and applied as antagonistic agents against a range of pest and plant pathogens. The utilization of microbial antagonistic bacteria, fungi, and yeast strains to manage plant disease or growth of phytopathogen during pre- or post-harvest management has been well elucidated [142,143]. Indeed, the addition of biosurfactants modulates the action mechanism, such as antibiosis, induced systemic resistance, competition, and parasitism of biocontrol agents [139].

*Pseudomonas* and *Bacillus* are the most common bacterial genera used for biosurfactant production. There are numerous reports available that showed their potency in biosurfactant production and their implication in successful phytopathogen management [138]. Varnier et al. [144] reported on Rhamnolipid, produced by the *Pseudomonas aeruginosa*, which enhanced the immune response of grapevine against the *Botrytis cinerea* after application. In addition, the application of surfactants inhibits the spore germination and mycelium growth of the pathogen. Kruijt et al. [145] reported the surfactants produced by *Pseudomonas putida*, which, after application, impede the growth of pathogen *Phytophthora capsici* in cucumber through zoospores lysis. Nielsen and Sorensen [146] reported that the surfactant cyclic lipopeptides produced by *Pseudomonas fluoresecens* have antifungal properties. Pernell et al. [147] evaluated the combined application of phenazines and rhamnolipid surfactant produced by *Pseudomonas aeruginosa* PNA1 strain. The application of both the metabolites showed a synergistic effect against the pathogen *Pythium splendens* of bean and *Pythium myriotylum* of cocoyam. In addition, substantial vacuolization and disintegration of *Pythium* hyphae were observed during microscopic analysis. Similarly, D’aes et al. [148] reported that phenazine and cyclic lipopeptide produced strain *Pseudomonas* CMR12a, which showed effective biocontrol potential against *Rhizoctonia* root rot on bean. Velho et al. [149] reported on the lipopeptide surfactant produced by *Bacillus*, having strong antagonistic activity against the pathogens *Aspergillus* sp., *Fusarium* sp., and *Biopolaris sorokiniana*.

Similarly, in another study, Akladious et al. [150] evaluated the biosurfactant produced by strain *Bacillus licheniformis*, which after application significantly controls the pathogen *Rhizoctonia solani,* the causal agent of root rot in faba beans. Hussain et al. [151] investigated biosurfactants produced by *Bacillus subtilis*, having bio-nematicidal activities against the pathogen *Meloidogyne incognita*, which is the causal agent of Root gall. Shalini et al. [152] investigated a glycolipid surfactant produced by the *Acinetobacter* sp., which showed antagonistic activity against *Xanthomonas oryzae* P.V. Oryzae XAV24. Haddad et al. [153] investigated surfactin biosurfactants produced by *Brevibacillus brevis*, having antibacterial and antifungal properties. Similarly, the endophytic strain *Burkholderia* sp. produced Glycolipid. The biosurfactant showed broad-spectrum antibacterial activity against the pathogens *Pseudomonas aeruginosa*, *E. coli*, and *Salmonella paratyphi* [154]. A detailed summary of the biosurfactants used in plant disease management has been described in Table 5. 

The application of surfactants acts differently during pathogen management. For example, Edosa et al. [161] investigated the action mechanism of some biosurfactants for insect pest management. The biosurfactant acts on the cell wall of the pests and causes significant damage due to dehydration. In a study, Yun et al. [162] investigated surfactin produced by *Bacillus amyloliquefaciens*, which affects the aphid cuticle after application, resulting in dehydration from the cuticle membrane, leading to dehydration and death. Similarly, Khedher et al. [163] observed vacuolization, necrosis, and basement membrane disintegration in the larval midgut of *Spodoptera littoralis* after histopathological examination of biosurfactant treatment. These reported biosurfactants and their application in plant disease management showed an excellent alternative to chemical pesticides, which are currently utilized in different parts of the world. However, the additional benefit of using microbial surfactant is the enhancement in plant growth and the providing nutrient source and favorable conditions for the native microflora that are essential for the plants to mitigate them from various biotic and abiotic stresses and for the degradation of toxic and hazardous environmental contaminants.

## 6. Biosurfactant Application in Pharmaceutical Industries

### 6.1. Antioxidant Properties of Biosurfactants

Nowadays, microbial surfactants have been used in the food and pharmaceutical industries as antioxidant agents. The antioxidants are the compounds need to neutralize the free radicals generated in the body during various physiological processes. The highly reactive nature of free radicals led to severe damage, known as oxidative stress or oxidative damage [164].The microbial origin source of biosurfactants can alter the physicochemical properties of surfaces. Thus, they can obviate the binding of other bacterial adhesions on the surface [165].

Similarly, they can also block the oxidative chain reaction flow by rendering the antioxidant activities [165]. Considering the biosurfactant characteristics, such as low toxicity and biodegradable, antimicrobial, and antioxidant properties, they gained significant industrial attention and are now preferred over the usage of synthetic antioxidants [166]. To overcome the toxic effects of synthetic surfactants and subside their side effects upon consumption, it is a prerequisite for finding natural and non-toxic bio-based products with potential antioxidant products [167]. Natural biosurfactants are one such natural product that is also reportedly capable of blocking the oxidative chain reaction flow by rendering the antioxidant activities. Hence, they also can effectively impede the elevation of reactive oxygen species (ROS) and reactive nitrogen species (RNS); hence, they could be highly useful for therapeutic purposes against cancer and the cure of heart-related diseases and neurodegenerative diseases [168]. Likewise, they were also highly instrumental in manufacturing probiotics, bio preservatives, and food ingredients [169].

Recently, several research groups explored various biosurfactants bestowed with excellent potential antioxidant properties from diverse sources. In addition to their potential antioxidant activity, some of the biosurfactants also displayed antimicrobial and antiproliferative activities [170,171,172]. In line with these findings, biosurfactant MB15, isolated from the non-pathogenic marine *Marinobacter litoralis* bacteria [171], was found to have no cytotoxic effect, but had a potent antioxidant and antimicrobial activity. Another report by Giri et al. assessed the antioxidant, antibiotic, and antiadhesive properties of the biosurfactant compounds isolated from *Bacillus subtilis* VSG4 and *Bacillus licheniformis* VS16. Their study revealed that the *Bacillus subtilis* VSG4 displayed better antioxidant activity than *Bacillus licheniformis* VS16 [173]. Meghna et al. also characterized a biosurfactant BS-LBL from *Lactobacillus casei*, and their experiment enlightened the efficient antioxidant, antimicrobial, and antiproliferative properties upon testing [172]. Likewise, Ohadi et al. [174] examined a biosurfactant obtained from *Acinetobacter junii*. They confirmed that the lipopeptide biosurfactant (LBS) from *A. junii* bestowed high antioxidant capacity with excellent wound healing ability in the mouse. Similar findings were also reported by other studies [170,175,176]. Collectively, the utilization and application of biosurfactants with antioxidant, antimicrobial, and antiproliferative substances will be a great addition to the products to safeguard consumer health benefits.

A few more reports have also consolidated the potent antioxidant and antimicrobial activities of biosurfactants lately. For example, Mouafao et al. identified and characterized a biosurfactant from *Lactobacillus casei* subsp. casei TM1B, which also confers efficient antioxidant and broad-spectrum antimicrobial activities convoyed with good emulsification and surface activities [177]. The biosurfactant MB588 from *Halobacillus karajiensis* showed a comparable antioxidant capacity as a positive control among all the isolates. It also showed higher antimicrobial activity; together, this suggests that the bacteria from extreme halophilic soils can also be helpful for the isolation of novel biosurfactants [178].

Similarly, another study by Abdollahi et al. compared two biosurfactants derived from two autochthonous strains for their antioxidant ability. Their study revealed that *Bacillus amyloliquefaciens NS6*-derived surfactin-natured biosurfactant displayed a more robust antioxidant capacity than *Pseudomonas aeruginosa* MN1-derived rhamnolipid-structured biosurfactant. However, they found that the rhamnolipid treated surfaces displayed higher antiadhesive and antibiofilm activities than surfactin-treated surfaces [165]. More examples of biosurfactants that possess antioxidant and antimicrobial activities [178,179] are listed in Table 6.

Few studies have explored the efficacy of natural biosurfactants that originated from a cost-effective substrate and compared their total antioxidant capacity (TAC) with a synthetic surfactant. Amaro da Silva et al. assed the TAC of a biosurfactant isolated from a low-cost substrate by *Candida bombicola* URM 3718 and compared it with a commonly used synthetic surfactant called Guar gum for its emulsification and total antioxidant capacity. Their study revealed that the biosurfactant predominantly displayed better antioxidant and emulsification ability than guar gum [166,174]. Other studies with different objectives also approved the need for biosurfactants to overcome the shortcomings of synthetic surfactants [180,181,182]. In summary, it is important to consider the numerous promising attributes of biosurfactants and their strong potential to elicit antioxidant activity on the detrimental reactive oxygen species and free oxygen radicals (H_2_O_2_, O_2_^_^, OH* and ^1^O_2_) from diverse sources. Because of natural origin, at the certain extent, biosurfactants also conferred antimicrobial activity and offer attractive opportunities to replace synthetic surfactants in the pharmaceutical, probiotic, and cosmetic industries. Thus, there is an urgent need for comprehensive characterization of each type of biosurfactant to be established to harness the best benefits and efficient application process.

### 6.2. Antimicrobial Properties of Biosurfactants

Multidrug resistance (MDR) is an emerging challenge for the growing world, especially in developing countries. However, in the recent past, antibiotic resistance has opened the door to search for alternative antimicrobial medicine to treat human ailments [183]. In this context, bacteriostatic and bactericidal, and biofilm disruption potential of biosurfcatants, make them ideal as an antimicrobial agent [184]. Numerous reports are available that showed the effectiveness of biosurfactants against different pathogens. For example, Foschi and others [185] reported antimicrobial effects against *Neisseria gonorrhoeae*. Similarly, Morais and others [186] observed against *Candida albicans,* Dusane and others [187] reported biofilm degradative behavior of rhamnolipid surfactant against *Bacillus pumilus*. 

However, biosurfactants produced by microbial strain act differentially during pathogen inhibition. For instance, Rhamnolipids possess activity through a permeabilizing effect, which leads to the disruption of the bacterial cell plasma membrane. The amphipathic nature of rhamnolipids binds with the charges of the bacterial cell membrane and changes their hydrophobicity. This prevents biofilm formation and makes the pathogen highly susceptible to the antimicrobial agent [188]. Several studies have suggested that rhamnolipids may act more effectively against Gram-positive bacteria than Gram-negative bacteria due to the absence of an outer membrane. The presence of the outer layer may exclude biosurfactant molecules [189]. However, the lipopolysaccharides biosurfactants attribute antimicrobial property via penetrating or damaging the lipid. The charge imbalance led to pore formation in the cell membrane lipids, which ultimately caused damage to or death of the pathogens, especially of Gram-negative bacteria [190].

In recent years, biosurfactants, such as lipopolysaccharides and glycolipids produced by microbial strains, have been used directly or indirectly as anticancer agents. Biosurfactants’ structural diversity and physio-chemical nature showed a broad-spectrum application during chemotherapy or drug delivery formulations. Currently, various reports show the effectiveness of glycolipids and lipopolysaccharides in controlling the proliferation of cancer cells and disrupting cell membranes through apoptosis pathways [191]. Zhao and others [192] reported the antitumor activity of lipopolysaccharides, composed of peptides and fatty acid chains. Dey and others [193] reported that Iturin synthesized by *Bacillus* strains inhibits the proliferation of MDA-MB-231 cancer cells.

## 7. Future Directions and Concluding Remarks

Biosurfactants are considered to be the multifunctional biomolecules of the 21st century, due to their broad application, ranging from daily life to industrial purposes. Currently, numerous microbial strains have been identified and screened for biosurfactant ability, and each day, some novel biosurfactant molecules have been identified and recovered. However, the biosurfactant’s fragile nature, lower stability, and high production cost appear as a critical barrier for frequent use in the industries. In the recent past, to reduce the production cost, various alternative sources of carbon or nitrogen, which are the essential requirements for microbial growth, have been utilized, and up to a certain extent, researchers have been successful. Nevertheless, the lower yield of biosurfactants, using alternative sources, is still a limiting factor. Therefore, there is a need for extensive study of certain factors, including biosynthesis pattern, growth, environmental conditions, and media composition for the large-scale production of biosurfactant molecules for the industrial uses and economic standpoint. 

Currently, rapid industrialization and anthropogenic behavior is leading to the deposition of toxic and hazardous contaminants in the soil, affecting environmental conditions and limiting agricultural production. Nowadays, biosurfactants have been broadly utilized to degrade the toxic, hazardous, and hydrophobic environmental contaminants and to improve the soil quality by maintaining the concentration of trace elements. The most commonly used surfactants are of chemical origin, and their uses in the agricultural fields can lead to food toxicity and can also adversely affect the natural microflora. However, the surfactants with a microbial origin have no such impact, and can even accelerate the growth of plants and microflora, which are required to degrade environmental contaminants. The selection of biosurfactants according to soil contaminants can enhance the soil quality better and in less time. For the sustainable growth of the rising global population, management of pre- and post-harvest losses of agricultural products is an immediate need. Currently, surfactants are mainly used as adjuvants or sometimes directly to the plant surface for phytopathogen management. However, there is still a need to explore the director adjuvants’ use of biosurfactants and their impact on the natural phytomicrobiome, residual level in fruits, and their impact on the physiological aspect of plants.

Moreover, there is also a need for the extensive investigation of biosurfactant molecules to explore novel antimicrobial agents, antioxidant molecules, and antiproliferative agents. This is not only cost-effective, but also protects the body from its toxic side effects. However for the treatment chronic diseases, cancer therapy and drug delivery using biosurfactants needs an extensive research. In addition, after the advancements in technology and resource materials, the high production cost, and the low yield of biosurfactants are still challenging tasks that need to be overcome.

## Figures and Tables

**Table 1:** Biosurfactant classification and some common examples.

	Types of Biosurfactants	Common Examples
Low molecular mass	Glycolipids	Rhamnolipids
		Sophorolipids
		Trehalose lipids
	Phospholipids	Phospholipids
		Corinomiocolic acid
		Fatty acids
	Lipopeptides	Surfactin
		Wisconsin
		Gramicidin
		Subtilisin
		Peptide lipid
		Lichenysin
High molecular mass	Polymeric	Liposan
		Emulsan
		Biodispersion
		Mannan-lipid protein
		Carbohydrate lipid-protein
	Particulate	Vesicles

**Table 2:** (**A**). Different types of biosurfactants produced from bacterial strains. (**B**). Different types of biosurfactants produced from fungal strains. (**C**). Different types of biosurfactants produced from yeast strains.

**(A). Different Types of Biosurfactants Produced from Bacterial Strains**				
**Bacterial Strains**	**Biosurfactants**	**Properties**	**Isolation Source**	**References**
*Pontibacter korlensis* strain SBK-47	Pontifactin	Surface-active, antimicrobial, and antibiofilm activities	Coastal waters of Karaikal, Puducherry, India	[23]
*Bacillus licheniformis*	Lipopeptides	Heat resistance and capacity to emulsify oils used in cosmetics	Deception Island (Antarctica)	[24]
*Paracoccus* sp. MJ9	Rhamnolipid	Enhance solubility of hydrophobic compounds	Jiaozhou Bay in Qingdao, Shandong Province	[25]
*Pseudomonas aeruginosa* UCP0992	Rhamnolipids	High emulsifying activities against different oils, capacity to remove hydrophobic contaminants, and did not show toxicity	Centre of Research in Environmental Sciences, Catholic University of Pernambuco, Brazil	[26]
*Pseudomonas aeruginosa* PA1	Rhamnolipid	Capacity to use as carbon sources	Oil production wastewater in the northeast of Brazil	[27]
*Pseudomonas desmolyticum* NCIM 2112	Rhamnolipid	Degradation of textile dye	National Center for Industrial Microorganisms (NCIM), Pune, India	[28]
*Serratia marcescens* SS-1	Serrawettins	Produces lipopeptide surfactants, having the capability to reduce surface tension	Taiwan	[29]
*Bacillus subtilis*	Cyclic lipopeptides	A significant reduction in the activities of acetylcholinesterase, a-carboxylesterase, and acid phosphatases	Namakkal and Tirunelveli district, Tamil Nadu, India	[30]
*Bacillus subtilis*	Pumilacidin	Antiviral activity against Herpes simplex virus 1 (HSV-1)	Tree trunk near lake Yamanaka, Japan	[31]
*Pseudomonas aeruginosa* S5	Glycolipid	Removal of polycyclic aromatic hydrocarbons	Supelco (Bellefonte, PA, USA)	[32]
*Pseudomonas protegens* F6	Orfamide A	Insecticidal against *Myzus persicae*	Soil from previously reported diesel oil-contaminated site	[33]
*Pseudomonas aeruginosa* DS9	Rhamnolipid	Antifungal agents against *F. sacchari* in pokkah boeng Disease	Lakota oil-field of Sivsagar district, Assam, India	[34]
*Pseudomonas fluorescens* BD5	Pseudofactin II	Antiadhesive activity and disinfectant	Freshwater from the Arctic Archipelago of Svalbard	[35]
*Bacillus* sp. BS3	Lipopeptide	Anticancer activity and antiviral properties	Solar salt works in Tamilnadu, India	[36]
*Pseudomonas aeruginosa*	Rhamnolipid	Enhanced oil recovery through anaerobic production of Rhamnolipid	Daqing oilfield-produced water	[37]
*Bacillus subtilis* A21	Lipopeptide	Removal of petroleum hydrocarbons, heavy metals	Adityapur Industrial Area, Jharkhand	[38]
*Rhodotorula bogoriensis*	Sophorolipid	Antimicrobial property against *Propionibacterium acnes*	American Type Culture Collection	[39]
**(B). Different Types of Biosurfactants Produced from Fungal Strains**				
**Fungi**	**Biosurfactants**	**Properties**	**Isolation Source**	**References**
*Candida utilis*	Emulsifiers	Emulsifiers	Culture collection from the Department of Antibiotics of the Universidade Federal de, Pernambuco, Brazil	[40]
*Candida lipolytica* UCP 0988	Lipopeptide	Not toxic against different vegetable seed	Culture collection of Nucleus of Research in Environmental Sciences, Catholic University of Pernambuco, Recife-PE, Brazil	[41]
*Penicillium chrysogenum* SNP5	Lipopeptide	Role in pharmaceuticals as well as in the petroleum and oil industry	Soil-contaminated grease waste	[42]
*Cunninghamella echinulata*	Complex Carbohydrate/protein/lipid	Reduce and increase the viscosity of hydrophobic substrates and their molecules	Caatinga soil of Pernambuco, Northeast of Brazil	[43]
*Candida Antarctica*	Mixtures of 4 mannosylerythritol lipids	Produced the lipids from different vegetable oils	Centraalbureau voor Schimmelcultures, the Netherlands	[44]
*Microsphaeropsis* sp.	Eremophilane derivative	Antimicrobial properties	Waters around the Caribbean Island of Dominica	[45]
*Yarrowia lipolytica* NCIM 3589	Bioemulsifier	Increased the hydrophobicity of the cells during the growth phase	Seawater near Mumbai, India	[46]
*Yarrowia lipolytica* IMUFRJ50682	Carbohydrate protein complex	Capable of stabilizing oil-in-water emulsions	Guanabara Bay in Rio de Janeiro	[47]
*Ustilago maydis*	Cellobiose lipids	Secreted cellobiose lipid having antifungal activity	-	[48]
*Torulopsis bombicola*	Sophorose lipid	Sophorose lipid fermentation	American Type Culture Collection	[49]
*Aspergillus ustus*	Glycolipoprote	Antimicrobial activity	Peninsular coast of India	[50]
*Candida bombicola* ATCC 22214	Sophorolipid	Used in low-end consumer products and household application	American Type Culture Collection	[51]
*Ustilago maydis* FBD12	Glycolipids	Antimicrobial activity	American Type Culture Collection	[52]
**(C). Different Types of Biosurfactants Produced from Yeast Strains**				
**Yeast**	**Biosurfactants**	**Properties**	**Isolation source**	**References**
*Starmerella bombicola*	Sophorolipids	Cytotoxic effect on MDA-MB-321 breast cancer cell line	Fungal Biodiversity Centre	[53]
*Torulopsis* *Petrophilum* ATCC 20225	Glycolipids	Protein emulsifier	American Type Culture Collection	[54]
*Kluyveromyces marxianus* FII 510700	Mannanoprotein	Source of emulsifier in the food industry	Culture Collection of the University of New South Wales, UNSW	[55]
*Pseudozyma aphids*, DSM 70725 and DSM 14930	Mannosylerythritol lipids	Foam formation	Deutsche Stammsammlung von Mikroorganismen und Zellkulturen (DSMZ), Braunschweig, Germany	[56]
*Pseudozyma tsukubaensis*	Glycolipid	Producing diastereomer MEL-B from vegetable oils	Leaves of Perilla frutescens on Ibaraki in Japan	[57]
*Saccharomyces cerevisiae* URM 6670	Glycolipid	Antioxidant activity and cytotoxic potential	Culture Collection of the Department of Antibiotics of the Federal University of Pernambuco (Brazil)	[58]
*Trichosporon asahii*	Sophorolipid	Efficient degrader of diesel oil, higher hydrophobicity, emulsification activity, and surface tension reduction	Petroleum hydrocarbon-contaminated soil in India	[59]
*Meyerozyma guilliermondii* YK32	Sophorolipid	Emulsification properties	Soil samples collected from hydrocarbon-polluted locations of Hisar, Haryana	[60]
*Rhodotorula babjevae* YS3	Sophorolipid	Antimicrobial activity	Agricultural field in Assam, Northeast India	[61]
*Pichia caribbica*	Xylolipid	Reduced the surface the tension of distilled water	Microbial type culture collection, India	[62]
*Candida ishiwadae* Y12	Monoacylglycerols: Glycolipid	Exhibited high surfactant activities	Plant material in Thailand	[63]

**Table 3:** The common substrates used in biosurfactant production and their yields.

Substrate	Conc. (gL^−1^)	Microorganisms	Yield (gL^−1^)	References
Glucose	40	*P. aeruginosa*	0.3	[72]
	40	*B. subtilis*	3.6	[73]
	40	*B. subtilis*	0.72	[74]
	30	*B. pumilus*	0.72	[75]
	20	*P. aeruginosa*	3.88	[76]
	10	*B. subtilis*	0.16	[77]
	-	*Pseudomonas* sp.	0.35	[78]
Sucrose	20	*P. putida*	1.30	[79]
Glucose and fructose	16.55	*B. subtilis*	0.93	[80]
Glucose + Yeast extract	1:3	*Bacillus* sp.	2.56	[81]
Glycerol + yeast extract	30:5	*P. aeruginosa*	2.7	[82]
Yeast extract	1	*P. taiwanensis*	1.12	[83]
Yeast extract	2	*Bacillus* sp.	2.5	[84]
NaNO_3_	0.2 M	*P. aeruginosa*	2.73	[85]
NaNO_3_	5	*B. subtilis*	1.12	[86]
Peptone	4	*Serratia marcescens*	1.2	[81]
NH_4_NO_3_	1	*P. fluorescens*	2	[87]

**Table 4:** The common alternative substrates and their impact on biosurfactant yield.

Microorganism	Alternative Media Source	Yield and Properties	References
*Bacillus subtilis* ATCC 6051	Brewery waste (trub)	The product yield of 100.76 mgL^−1^	[96]
*Bacillus subtilis* PC	Sugar cane vinasse	Able to reduce surface tension 32 mN m^−1^ and the E24 to 51.10%.	[97]
*Bacillus subtilis*	Corn steep liquor	Biosurfactant yields 1.3 gL^−1^; the different yields increased (up to 4.1, 4.4, and 3.5 g/L for iron, manganese, and magnesium supplements, respectively). However, at the optimum concentration, the yield of these three metals increased up to 4.8 gL^−1^.	[98]
*Bacillus subtilis* MTCC 2423	Rice mill polishing residue	Surfactin yield 4.17 g kg^−1^ residue	[99]
*Bacillus licheniformis* AL1.1	Molasses	Lichenysin yield of 3·2 gL^−1^	[100]
*Bacillus pseudomycoides*	Soybean oil waste	C.M.C. of lipopeptide 56 mgL^−1^ and able to reduce the surface tension of water from 71.6 mN m^−1^ to 30.2 mN m^−1^	[101]
*Bacillus subtilis* DSM 3256	Two-phase olive mill waste	Surfactin yields 0.068 g g^−1^, and the surface tension of the culture medium is reduced to 30.1 ± 0.9 mN m^−1^.	[102]
*Bacillus subtilis*	Rapeseed cake	Surfactin analogues	[103]
*Bacillus amyloliquefaciens*	Distillers’ grains	Surfactin yield 1.04 gL^−1^	[104]
*Bacillus nealsonii* S2MT	Glycerol 2% (*v*/*v*) and NH4NO3 0.1% (*w*/*v*)	The maximum biosurfactant yield was 1300 mg/L and reduced the surface tension (34.15 ± 0.6 mN/m). Additionally, highly stable at environmental factors such as salinity, pH and temperature variations.	[105]
*Staphylococcus* sp.	Residual frying oil, expired milk	The C.M.C. of the purified lipopeptides was 65–750 mg/L, depending upon carbon source. Additionally, it was stable within a broad range of pH, temperature, and salinity values.	[106]
*Halomonas venusta* PHKT	Glycerol	Surfactin, Pumilacidin, and Bios-PHKT have a critical micelle concentration (C.M.C.) of 125 mgL^−1^ and showed a high steadiness against a broad spectrum of salinity (0–120 gL^−1^ NaCl), temperature (4–121 °C), and pH values (2–12).	[107]
*Rhodotorula* sp.	Olive oil mills	Potent biosurfactant producer with E24 = 69% and a significant reduction in S.T. from 72 to 35 mN m^−1^. In addition, it showed stability over a wide range of pH (2–12), temperature (4–100 °C), and salinity values (1–10%).	[108]
*Volvariella volvacea*	Edible paddy straw mushroom	Biosurfactant effectively showed a reduction in the surface tension, emulsification index, and oil spreading activity of 35.15 dyne/cm, 80%, and 11 cm, respectively.	[109]

**Table 5:** The common biosurfactants used in plant disease management.

Microorganism	Biosurfactant	Properties	Reference
*Pseudomonas* sp. EP-3	Rhamnolipid	Insecticidal activity	[155]
*Pseudomonasaeruginosa* PAO1	Rhamnolipid	Biofilm formation	[156]
*Pseudomonas aeruginosa*	Rhamnolipids	Control of *Phytophthora cryptogea*	[157]
*Pseudomonas aeruginosa*	Rhamnolipids	Resistance to *Botrytis cinerea* in grapevine	[144]
*Pseudomonas putida*	Biosurfactants	Zoospores of the oomycete pathogen *Phytophthora capsici*	[145]
*Pseudomonas koreensis*	Biosurfactant	Late blight on potato	[158]
*Acinetobacter* sp. *ACMS25*	Glycolipid	Biocontrol of Xanthomonas oryzae	[152]
*Burkholderia* sp. WYAT7	Glycolipid	Antibacterial and ant- biofilm potentials	[154]
*Bacillus licheniformis*	Biosurfactant	Biocontrol of *Rhizoctonia solani* causing root rot in faba bean	[150]
*Pseudomonas* CMR12a	Lipopeptides	Biological control of Rhizoctonia root rot on bean	[148]
*Brevibacillus brevis*	Lipopeptides	Antibacterial and Antifungal properties	[153]
*Bacillus* sp.	Lipopeptides	Growth inhibition of *Fusarium* spp., *Aspergillus* spp., and *Biopolaris sorokiniana*	[149]
*Bacillus subtilis* R14	Lipopeptide	Antimicrobial activity	[159]
*Bacillus subtilis*	Lipopeptides Iturin A, fengycin, and surfactin	Colletotrichum gloeosporioides, the causative agent for anthracnose on papaya leaves	[160]

**Table 6:** Antioxidant properties of biosurfactants.

Source	Chemical Nature of Biosurfactant	Antioxidant Activity Assessment	Antioxidant	Antibacterial	Antiproliferative	Reference
*Lactobacillus casei* subsp. casei TM1B	Rhamnolipid-like biosurfactant	DPPH (1-diphenyl-2-picrylhydrazyl) assay, ABTS (2.2′-azino-bis(3-ethylbenzothiazoline-6-sulfonic acid) assay	yes	yes	not tested	[177]
*Pseudomonas aeruginosa* MN1	Rhamnolipid	FRAP and DPPH assay	yes	yes	not tested	[165]
*Bacillus amyloliquefaciens* NS6	Surfactin	Ferric reducing antioxidant power (FRAP) and DPPH assay	yes	yes	not tested	[165]
*Marinobacter litoralis* MB15	Rhamnolipid	DPPH assay	yes	yes	yes	[171]
*Halomonas elongata*, *Halobacillus karajiensis* and *Alkalibacillus almallahensis*	Glycolipid	DPPH assay	yes	yes	not tested	[178]
*Bacillus subtilis* VSG4	Lipopeptide	DPPH assay	yes	yes	not tested	[173]
*Bacillus licheniformis* VS16	Phospholipopeptide	DPPH assay	yes	yes	not tested	[173]
*Lactobacillus casei* (BS-LBl)	Not mentioned	DPPH assay	yes	yes	yes	[172]
*Acinetobacter junii* B6	Lipopeptide	DPPH and FRAP assay	yes	yes	not tested	[174]
*Bifidobacterium bifidum* WBIN03 and *Lactobacillus plantarum* R315	Exo polysaccharides	DPPH assay and superoxide and hydroxy radical estimation	yes	yes	not tested	[180]
*Bacillus methylotrophicus* DCS1	Lipopeptide	DPPH assay	yes	yes	not tested	[179]
*Pseudozyma hubeiensis*	Mannosylerythritol lipids	DPPH assay	yes	not tested	yes	[175]
*Bacillus subtilis* SPB1	Lipopeptide	DPPH assay	yes	not tested	yes	[176]
*Bacillus cereus* MMIC	Lipopeptide	DPPH assay	yes	yes	yes	[170]

## Data Availability

Data is available within the article.
